# Migrating songbirds on stopover prepare for, and recover from, oxidative challenges posed by long-distance flight

**DOI:** 10.1002/ece3.1601

**Published:** 2015-07-14

**Authors:** Megan M Skrip, Ulf Bauchinger, Wolfgang Goymann, Leonida Fusani, Massimiliano Cardinale, Rebecca R Alan, Scott R McWilliams

**Affiliations:** 1 Department of Natural Resources Science, University of Rhode IslandKingston, Rhode Island; 2 Institute of Environmental Sciences, Jagiellonian UniversityKraków, Poland; 3 Max-Planck-Institut für Ornithologie, Abteilung für VerhaltensneurobiolgieSeewiesen, Germany; 4 Department of Life Sciences and Biotechnology, University of FerraraFerrara, Italy; 5 Department of Aquatic Resources, Marine Research Institute, Swedish University of Agricultural SciencesLysekil, Sweden

**Keywords:** Antioxidant capacity, condition dependency, ecological barriers, lipid oxidation, long-distance flight, migration strategy

## Abstract

Managing oxidative stress is an important physiological function for all aerobic organisms, particularly during periods of prolonged high metabolic activity, such as long-distance migration across ecological barriers. However, no previous study has investigated the oxidative status of birds at different stages of migration and whether that oxidative status depends on the condition of the birds. In this study, we compared (1) energy stores and circulating oxidative status measures in (a) two species of Neotropical migrants with differing migration strategies that were sampled at an autumn stopover site before an ecological barrier; and (b) a species of trans-Saharan migrant sampled at a spring stopover site after crossing an ecological barrier; and (2) circulating oxidative measures and indicators of fat metabolism in a trans-Saharan migrant after stopovers of varying duration (0–8 nights), based on recapture records. We found fat stores to be positively correlated with circulating antioxidant capacity in Blackpoll Warblers and Red-eyed Vireos preparing for fall migration on Block Island, USA, but uncorrelated in Garden Warblers on the island of Ponza, Italy, after a spring crossing of the Sahara Desert and Mediterranean Sea. In all circumstances, fat stores were positively correlated with circulating lipid oxidation levels. Among Garden Warblers on the island of Ponza, fat anabolism increased with stopover duration while oxidative damage levels decreased. Our study provides evidence that birds build antioxidant capacity as they build fat stores at stopover sites before long flights, but does not support the idea that antioxidant stores remain elevated in birds with high fuel levels after an ecological barrier. Our results further suggest that lipid oxidation may be an inescapable hazard of using fats as the primary fuel for flight. Yet, we also show that birds on stopover are capable of recovering from the oxidative damage they have accrued during migration, as lipid oxidation levels decrease with time on stopover. Thus, the physiological strategy of migrating songbirds may be to build prophylactic antioxidant capacity in concert with fuel stores at stopover sites before a long-distance flight, and then repair oxidative damage while refueling at stopover sites after long-distance flight.

## Introduction

Managing oxidative stress is an important physiological function for all aerobic organisms, particularly during periods of high metabolic activity such as exercise, when mitochondria increase generation of harmful pro-oxidants (Niess 2005). Long-distance endurance exercise such as migratory flight poses an especially high oxidative challenge for bird species, particularly those that cross ecological barriers with little opportunity for rest or recovery. Migrating songbirds presumably respond to an increased potential for oxidative stress by up-regulating protective endogenous systems or accumulating dietary antioxidants at stopover sites (Costantini et al. 2007; Alan et al. 2013; Bolser et al. 2013; Jenni-Eiermann et al. 2014). Yet, no studies to date have investigated the oxidative status of birds at different stages of migration, at stopover sites before and after ecological barriers, and whether that oxidative status depends on the condition of the birds.

During migration, birds spend considerable time and energy at stopover sites, where they accumulate the necessary nutritional resources for completing the next leg of their journeys. At stopover sites, migratory songbirds build their fat stores in relation to the distance they must fly to reach their destination, and to meet energetic challenges (e.g., storms or barriers) they face along the way (Berthold 1993, 1996; Schaub and Jenni 2000; Fransson et al. 2008; Schaub et al. 2008). Songbirds then use stopover sites after long flights to recover fat, lean, and/or water mass they have lost during the course of those flights (e.g., Leberg et al. 1996). Presumably, songbirds facing long flights or ecological barriers must also build up adequate protection, in the form of antioxidants, to prevent the oxidative damage possible from their own metabolism; that is, they should build and use antioxidant protection as they build and use their fat stores. Yet, studies of this phenomenon are lacking. Birds might also use stopover sites after long flights to replace exhausted antioxidants and/or purge oxidative damage. However, no studies have tracked changes in oxidative status in relation to stopover duration.

In this study, we examined the oxidative status of three species of songbirds, in relation to their fuel stores, under several migration scenarios and contexts: (1) during the early stage of fall migration when Blackpoll Warblers (*Setophaga striata* Forster 1772) and Red-eyed Vireos (*Vireo olivaceus* Linnaeus 1766) rest and refuel at an island stopover site off the coast of southern New England, USA, before they head south over the Atlantic Ocean for thousands of kilometers of sustained flight or travel along the western Atlantic coast; and (2) during the late stage of spring migration when Garden Warblers (*Sylvia borin* Boddaert 1783) rest and refuel at a stopover site off the coast of Italy, immediately after flying hundreds of kilometers over the Mediterranean Sea from winter quarters in sub-Saharan Africa. We evaluated the following hypotheses and predictions: (Hypothesis 1) songbirds prepare for a migratory flight by building both their nonenzymatic antioxidant capacity and fat stores, as they likely consume foods rich both in antioxidants and fat, and therefore, these measures will be correlated in birds captured prior to departure on a long migratory flight; (Hypothesis 2) birds use both their energy stores and antioxidant stores during migration, and therefore, these measures will be correlated at a stopover site after an ecological barrier; (Hypothesis 3) birds with greater fat stores have a higher risk of oxidative damage, given that fat deposits are vulnerable to pro-oxidants generated during metabolism, and therefore, circulating lipid peroxidation levels will correlate with fat stores in all circumstances; (Hypothesis 4) birds rebuild both their antioxidant capacity and fat stores on stopover after an ecological barrier, and therefore, newly landed birds will have lower measures of both antioxidant capacity and fat anabolism than birds residing longer on stopover, and both measures will correlate with time on stopover.

## Materials and methods

### Field techniques – Block Island, RI, USA

We used mist nets to capture Blackpoll Warblers and Red-eyed Vireos on Block Island, Rhode Island, USA, between 5 September and 31 October 2012 and 2013. Block Island is a small glacially deposited island 15.5 km off the southern coast of Rhode Island (41°13′N, 71°33′W) and an important stopover site for millions of migrating songbirds each autumn (Reinert et al. 2002). Blackpoll Warblers breed in the Canadian boreal forest and Alaska and then gather on the northern Atlantic coast of the United States in autumn for a trans-oceanic migration to South America (Baird 1999; Vuilleumier 2009; DeLuca et al. 2015). This species is therefore unique among the passerines that migrate along the western Atlantic coast, in that it accumulates considerable fat stores (doubling a lean mass of 9–11 g) in preparation for long-duration flights over the ocean to wintering grounds in northern South America, rather than take routes offering a number of coastal or inland stopover sites for refueling (Baird 1999). Unlike the Blackpoll Warbler, the Red-eyed Vireo travels over land to its wintering areas and accumulates less fat in autumn (ca. 5 g above lean mass; Cimprich et al. 2000); this species has the longest migration of the North American vireos, breeding in northeastern North America and traveling in autumn through Central America and the Caribbean to the Amazon basin (Harris 2009). Thus, Red-eyed Vireos stopping over on Block Island during fall are preparing for the next leg of their overland southbound journey like most Neotropical migrants and may therefore be expected to have lower fat and antioxidant levels than Blackpoll Warblers.

Mist nets were operated on Block Island from 30 min before dawn until no later than 1600 local time. Within 1 h of a bird entering the mist net, we measured subcutaneous fat score on a 0–8 scale (Kaiser 1993) and body mass to 0.1 g. For 56 hatch-year Blackpoll Warblers and 63 hatch-year Red-eyed Vireos, we drew a ca. 150-*μ*L blood sample to measure nonenzymatic plasma antioxidant capacity and plasma oxidative damage. All blood samples were centrifuged within 30 min of capture for 6 min at 5000 rpm, and the plasma was flash frozen under liquid nitrogen. Plasma samples were later stored in the laboratory at −80°C until analysis.

The range of fat scores observed in Red-eyed Vireos was expected to be smaller than in Blackpoll Warblers, and so we also quantitatively measured fat mass of Red-eyed Vireos using the deuterium dilution method (Karasov and Pinshow 1998; McWilliams and Whitman 2013). After drawing a blood sample for plasma metabolites, we injected each bird with 50 *μ*L of 99% deuterium-enriched water (D_2_O) and drew a second 150-*μ*L blood sample after a 60-min equilibration period in a cloth bag (see McWilliams and Whitman 2013 for details). These blood samples were flame-sealed and stored at 4°C until later analysis in the laboratory (described below). All work on Block Island was approved by the University of Rhode Island’s Institutional Animal Care and Use Committee (IACUC # AN09-09-008).

### Field techniques – Ponza, Italy

We used mist nets to capture Garden Warblers on Ponza, Italy, between 10 and 19 May 2012. Ponza is a small volcanic island 50 km off the Tyrrhenian coast of Italy (40°55′N, 12°58′E), where thousands of songbirds on broad-front northward migration stopover after flying long durations (≥10 h) from Africa in spring (Grattarola et al. 1999). The Garden Warbler breeds in west temperate Eurasia and winters in sub-Saharan Africa, crossing the Sahara Desert and Mediterranean Sea during fall and spring migrations; this species has been the subject of many migration studies (Berthold 1993; Grattarola et al. 1999; Biebach et al. 2000; Bauchinger et al. 2005), including those recently focused on stopover physiology on or near Ponza (Costantini et al. 2007; Fusani et al. 2009, 2011; Goymann et al. 2010).

Mist nets were operated on Ponza from 30 min before dawn until no later than 1 h after sunset. Within 1 h of capture, we measured subcutaneous fat score on a 0–8 scale (Kaiser 1993), size of the pectoral muscles scored on a 0–3 scale (Gosler 1991), and body mass to 0.1 g. For 129 newly captured individuals, we drew a ca. 150-*μ*L blood sample within 3 min of a bird entering the mist net to measure the following parameters: nonenzymatic antioxidant capacity, oxidative damage, uric acid, *β*-hydroxybutyrate, triglycerides, and nonesterified fatty acids. All blood samples were centrifuged in the field for 6 min at 5000 rpm, and the plasma was flash frozen under liquid nitrogen within 15 min of capture. Plasma samples were later stored in the laboratory at −80°C until analysis. For 45 of these 129 birds, we also measured fat mass using the deuterium dilution method, as described above. An additional 26 Garden Warblers were placed in cloth handling bags for between 20 and 162 min (mean = 95 min) before blood sampling, to evaluate changes in blood metabolites with time.

We stratified sampling to include Garden Warblers sampled on both high-capture and low-capture days. On Ponza and other islands in the Mediterranean Sea, high-capture days are characterized by large influxes of birds on broad-front migration, which makes it more likely that birds on these days just arrived to the island after a ≥ 10-h flight. We conservatively defined a high-capture day as one with >1000 total captures, >90% of captures occurring after noon (marking the arrival of birds from the African coast), and capture rates after noon >100 birds per hour. We conservatively defined a low-capture day as one with equivalent netting effort but <200 total captures, <50% of captures occurring after noon, and capture rates after noon <30 birds per hour.

We also drew blood samples for circulating metabolites in the manner described above for 24 individuals that had been previously captured during normal ringing operations on Ponza, and that were opportunistically recaptured between 1 and 192 h after initial capture. These individuals constituted a recaptures dataset with a known minimum duration of stopover. Two birds were blood-sampled twice, on two capture occasions, and therefore provided information on intra-individual trends in blood metabolites. All work on Ponza was performed under permission number A00785 as of 8 February 2013 from the government of the Regione Lazio according to Italian law.

### Measurement of plasma metabolites

We used commercial kits modified for small volumes to perform metabolite assays for each of the 56 Blackpoll Warblers and 63 Red-eyed Vireos on Block Island, and the 129 newly captured, 26 held-in-bag, and 24 recaptured Garden Warblers on Ponza. Sample sizes listed in the results are not equal across all assays because not enough plasma was available to complete all assays for each individual. We performed all metabolite assays at the University of Rhode Island using a microplate spectrophotometer (Biotek Powerwave 340, Winooski, VT). We ran samples in duplicate unless a coefficient of variation >15% was observed, in which case we ran a third replicate if sufficient plasma was available. We determined antioxidant capacity as the ability of a plasma sample to neutralize an oxidizing assault of hypochlorous acid, using the OXY-Adsorbent Test (concentration unit = mmol/L of HClO neutralized; Diacron International, Grosseto, Italy); and measured oxidative damage as the presence of circulating hydroperoxides, which include products of lipid oxidation, using the d-ROMs test (concentration unit = mmol/L H_2_O_2_ equivalents; Diacron International, Grosseto, Italy; see Costantini et al. 2007 for further details). We chose the OXY and d-ROMs tests as general whole-animal markers of circulating oxidative status and to facilitate comparison of results with previous studies. We anticipated that OXY, as a measure of plasma nonenzymatic antioxidant capacity, would be particularly relevant in birds acquiring dietary antioxidants (Beaulieu and Schaefer 2014) on stopover as they consume fats; the d-ROMs test provides an index of damage to fats and has been shown recently to covary with circulating triglyceride levels (Pérez-Rodriguez et al. 2015).

We used endpoint assays to measure uric acid (concentration unit = mmol/L; Teco Diagnostics Anaheim, CA), triglycerides (concentration unit = mg/mL; Sigma-Aldrich Corporation, St. Louis, MO), and nonesterified fatty acids (concentration unit = mEq/L; Wako Diagnostics, Richmond, VA), and a kinetic assay to measure *β*-hydroxybutyrate (concentration unit = mmol/L; R-Biopharm, Darmstadt, Germany; Pierce et al. 2005; Smith et al. 2007; Smith and McWilliams 2009). Such metabolite assays are often used to track lipid uptake and deposition during feeding (circulating triglycerides), fat breakdown during exercise or fasting (*β*-hydroxybutyrate and nonesterified fatty acids), and protein catabolism (uric acid, which also functions as an endogenous antioxidant) in songbirds (Jenni-Eiermann et al. 2002; Pierce et al. 2005; Smith and McWilliams 2009, 2010).

### Determination of fat mass via deuterium dilution

We estimated fat mass of 62 Red-eyed Vireos and 45 Garden Warblers injected with deuterium, given the volume of deuterium injected, the measured concentration of deuterium in plasma, and the predictive models and approach described in McWilliams and Whitman (2013). Briefly, we microdistilled the water from each flame-sealed blood sample and used infrared spectrophotometry to measure deuterium concentration (Karasov et al. 1988). Deuterium concentration was measured at the University of Rhode Island using a FT-IR Spectrometer with a Universal ATR Sampling Accessory, with Spectrum and Spectrum Quant software (PerkinElmer, Waltham, MA). We verified the mass of D_2_O injected into birds (0.055 g) by weighing capillary tubes of the same injection volume, randomly selected from samples taken in the field. We then used this injection mass, the molar masses of D_2_O (20 g/mol) and unlabeled H_2_O (18 g/mol), and the deuterium enrichment of distilled blood water as determined by spectrophotometry to calculate estimated water space (Karasov and Pinshow 1998; McWilliams and Whitman 2013). We excluded from the dataset any values of estimated water space that were either <50 or >80% of body mass, given that these values are biologically unreasonable (McWilliams and Whitman 2013) and suggest that the samples were compromised. Fifteen Red-eyed Vireos and seven Garden Warblers were excluded from the dataset because their values were >80%, leaving 47 Red-eyed Vireo samples and 38 Garden Warbler samples for statistical analysis. We used the interspecific model in McWilliams and Whitman (2013) to estimate fat mass of Garden Warblers given estimated water space and measured body mass, and the model for Red-eyed Vireo in McWilliams and Whitman (2013) to estimate fat mass of Red-eyed Vireos given estimated water space and measured body mass.

### Statistical analyses

All statistical analyses were performed with SAS 9.4 software (SAS Institute 2014). We used nonparametric Spearman coefficients for all correlation analyses, given that variables of interest were not all normally distributed, even when transformed.

#### Newly captured Blackpoll Warblers, Red-eyed Vireos, and Garden Warblers

We used the CORR procedure to perform correlation analysis, to compare fat score, fat mass determined by deuterium dilution, and muscle score with antioxidant capacity and oxidative damage levels in newly captured birds, by species. We used the MIXED procedure to perform ANCOVAs (covariate = fat score) and to compare antioxidant capacity and oxidative damage between Blackpoll Warblers and Red-eyed Vireos (treatment = species); we estimated variances separately for each species, given that the assumption of homogenous residual variance was violated. We therefore report effect tests with adjusted degrees of freedom, using the Kenward–Roger adjustment, to accommodate this more complicated covariance structure (Kenward and Roger 1997; Schaalje et al. 2002). For the 26 Garden Warblers held in cloth bags, we used correlation analysis to compare plasma metabolites and time before sampling.

#### Garden Warblers captured on high-capture vs. low-capture days

Although we captured 129 previously unbanded Garden Warblers, the amount of time that they had spent on the island before capture could not be determined with certainty for most individuals. However, if we assume that the probability of capturing a newly arrived bird is higher on a high-capture day, then a comparison of the 28 high-capture-day birds with the 19 low-capture-day birds may indicate how plasma metabolites change with time at a stopover site. We used t-tests to compare the following variables in birds caught on high-capture and low-capture days: antioxidant capacity, oxidative damage, uric acid, *β*-hydroxybutyrate (log-transformed to achieve a normal distribution), triglycerides (also log-transformed), and nonesterified fatty acids.

#### Recaptured Garden Warblers

We used data from Garden Warblers with known minimum durations of stay on the island (from recapture records) to assess the correlation between elapsed time and blood metabolites. Two birds were sampled twice, on two capture occasions. To ensure data independence, we used only the first measurements from these two individuals for the correlation analyses, but we report both measurements in the Results section.

## Results

### Newly captured Blackpoll Warblers, Red-eyed Vireos, and Garden Warblers

#### Block Island, USA

**Figure 1:**
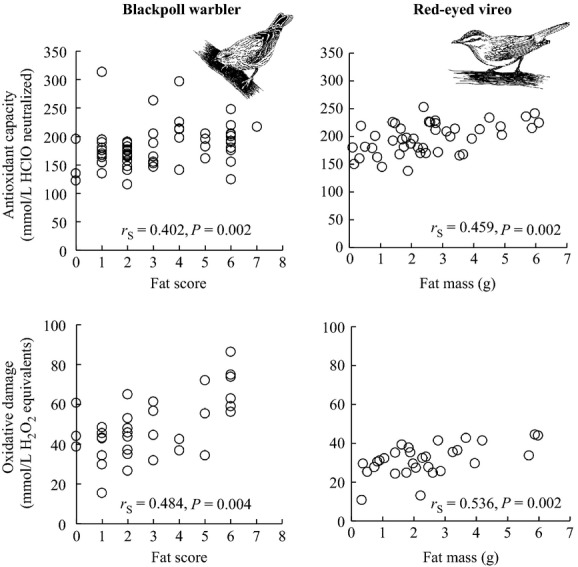
Fat stores were correlated with both antioxidant capacity and oxidative damage among Blackpoll Warblers (left) and Red-eyed Vireos (right) in autumn on Block Island, USA. Although fat score was measured in Red-eyed Vireos, it was less strongly correlated with oxidative measures than fat mass.

Fat score was positively correlated with both antioxidant capacity (*n* = 57) and oxidative damage (*n* = 33) among Blackpoll Warblers stopping over during fall migration on Block Island (Fig.1). Among Red-eyed Vireos, fat mass was positively correlated with antioxidant capacity (*n* = 45) and oxidative damage (*n* = 30) (Fig.1); fat score was also positively correlated with antioxidant capacity (*n* = 62, *r*_S_ = 0.357, *P *=* *0.004), but not with oxidative damage (*n* = 40, *r*_S_ = 0.275, *P *=* *0.086). Fat mass and fat score of Red-eyed Vireos were positively correlated (*n* = 47, *r*_S_ = 0.60, *P *<* *0.001). In both species, antioxidant capacity and oxidative damage were not significantly correlated (Blackpoll Warbler *n* = 33, *r*_S_ = 0.295, *P *=* *0.095; Red-eyed Vireo *n* = 41, *r*_S_ = 0.222, *P *=* *0.162).

**Figure 2:**
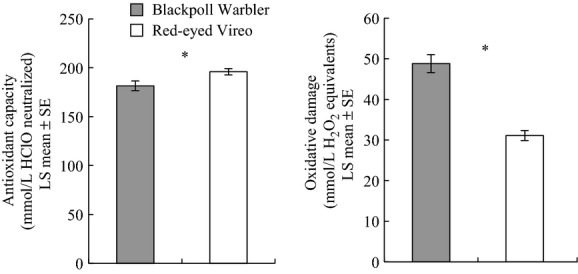
Antioxidant capacity (ANCOVA, with fat score as covariate) was lower in Blackpoll Warblers than Red-eyed Vireos, and oxidative damage was higher in Blackpoll Warblers than Red-eyed Vireos in autumn on Block Island, USA. Asterisks indicate a significant difference between species (*P* < 0.05).

We performed ANCOVA (covariate = fat score) to compare antioxidant capacity between Blackpoll Warblers and Red-eyed Vireos (treatment = species); the fat score * species interaction was insignificant and therefore removed from the model (*F*_1,107_ = 1.58; *P *=* *0.21). Blackpoll Warblers (*n* = 57) had significantly lower antioxidant capacity (LS mean ± SE, 181.44 ± 4.88 mmol/L HClO neutralized) than Red-eyed Vireos (*n* = 62, 195.90 ± 3.31) (species *F*_1,98.4_ = 5.94, *P *=* *0.016; fat score *F*_1,102_ = 12.53, *P *=* *0.0006; Fig.2). We performed ANCOVA to also compare oxidative damage between species; the fat score * species interaction was insignificant and therefore removed from the model (*F*_1,68.7_ = 0.47; *P *=* *0.50). Blackpoll Warblers (*n* = 33) had significantly higher circulating lipid peroxidation levels (48.82 ± 2.23 mmol/L H_2_O_2_ equivalents) than Red-eyed Vireos (*n* = 40, 31.10 ± 1.23) (species *F*_1,49.8_ = 48.31, *P *<* *0.0001; fat score *F*_1,65.2_ = 20.17, *P *<* *0.0001; Fig.2).

#### Ponza, Italy

**Figure 3:**
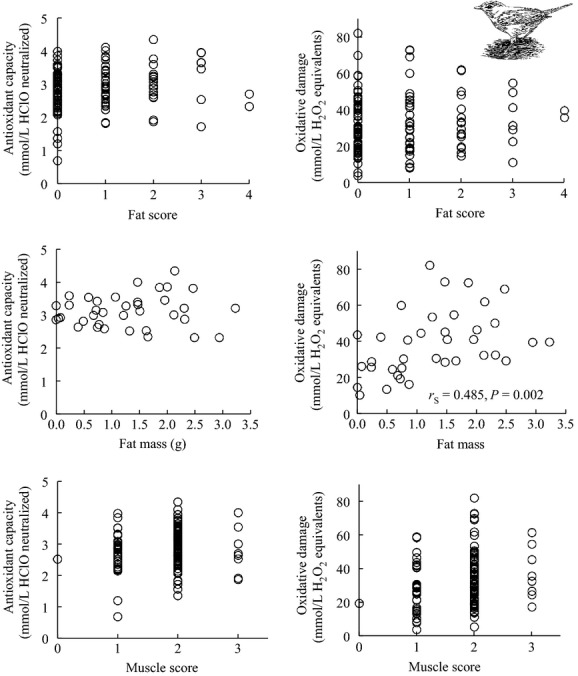
Only fat mass was correlated with only oxidative damage among newly captured Garden Warblers in spring on Ponza, Italy; fat score and muscle score were uncorrelated with oxidative measures.

Fat score was not correlated with either antioxidant capacity (*n* = 123, *r*_S_ = 0.118, *P *=* *0.195) or oxidative damage (*n* = 123, *r*_S_ = 0.118, *P *=* *0.195) among migrating Garden Warblers newly captured on spring stopover (Fig.3). Antioxidant capacity and oxidative damage were uncorrelated (*n* = 129, *r*_S_ = 0.067, *P *=* *0.452). Fat mass was uncorrelated with antioxidant capacity (*n* = 37, *r*_S_ = 0.060, *P *=* *0.726), but positively correlated with oxidative damage (*n* = 37, *r*_S_ = 0.485, *P *=* *0.002) (Fig.3). Muscle score was not correlated with either antioxidant capacity (*n* = 123, *r*_S_ = 0.090, *P *=* *0.324) or with oxidative damage (*n* = 123, *r*_S_ = 0.164, *P *=* *0.070) (Fig.3). Fat mass and fat score were positively correlated (*n* = 38, *r*_S_ = 0.48, *P *=* *0.002), and when we excluded three questionable points with a fat score of zero and ≥2.0 g of fat mass, the correlation improved (*n* = 35, *r*_S_ = 0.68, *P *<* *0.001). However, excluding these points did not significantly improve the correlation between fat score and oxidative measures.

Among the 26 Garden Warblers held in cloth bags before blood sampling, there was no significant correlation between time and oxidative measures (antioxidant capacity and oxidative damage; *n* = 26, *r*_S_ < ¦0.25¦, *P *>* *0.3) or between time and most of the plasma metabolites (uric acid, triglycerides, nonesterified fatty acids; *n* ≥ 21, *r*_S_ < ¦0.35¦, *P *>* *0.2). We did find that *β*-hydroxybutyrate increased with time before sampling (*n* = 23, *r*_S_ = 0.43, *P *=* *0.039), clearly a consequence of forced fasting while in handling bags.

### Garden Warblers captured on high-capture vs. low-capture days

**Figure 4:**
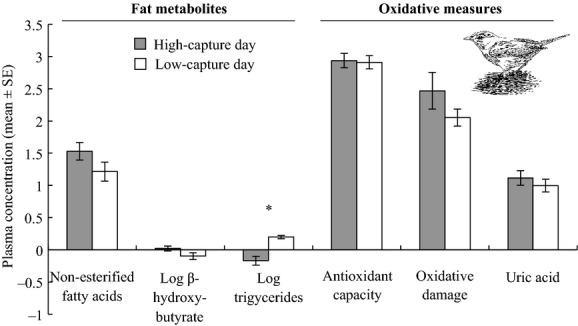
Newly captured Garden Warblers sampled on high-capture days (filled bars) had similar oxidative measures to birds sampled on low-capture days (open bars), but dissimilar plasma fat metabolites, particularly triglycerides. See Table1 for t-statistics and *P*-values, and Methods for concentration units for each plasma measure. Oxidative damage values displayed here are divided by 10 to fit on the same axis as other measures.

Newly captured Garden Warblers sampled on high-capture days had lower plasma levels of triglycerides than low-capture-day birds (Fig.4, Table1). However, they did not significantly differ from birds sampled on low-capture days in their levels of nonesterified fatty acids, *β*-hydroxybutyrate, antioxidant capacity, oxidative damage, or uric acid. Fat scores of Garden Warblers sampled on high- and low-capture days were mostly 0–1 (94% of low-capture day birds and 71% of high-capture-day birds).

**Table 1:** Comparison of plasma measures of oxidative state and fat metabolism between Garden Warblers captured on high-capture or low-capture days. High-capture days are characterized by large influxes of birds on broad-front migration, which makes it more likely that birds on these days had just arrived to the island after a ≥ 10-h flight

N		Type of assay	Plasma measure	df	*t*-value	*P*-value
High-capture	Low-capture					
28	19	Oxidative state	Antioxidant capacity^1^	45	−0.17	0.869
28	19	Oxidative state	Oxidative damage^2^	37.3	−1.32	0.193
28	19	Oxidative state	Uric acid^1^	45	−0.73	0.471
28	19	Fat metabolite	log *β*-hydroxybutyrate^1^	45	−1.82	0.076
25	18	Fat metabolite	log triglycerides^2^	30.4	5.08	<0.0001
23	17	Fat metabolite	Nonesterified fatty acids^1^	38	−1.55	0.129

1 variance calculation used = pooled.

2 variance calculation used = Satterthwaite.

### Recaptured Garden Warblers

**Figure 5:**
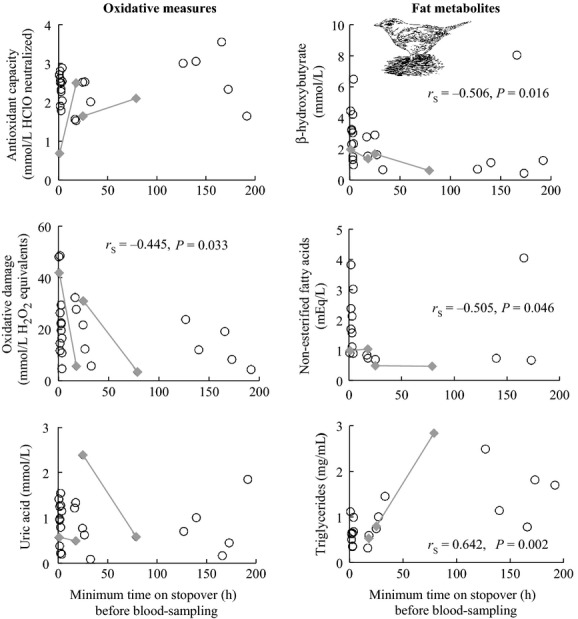
Levels of six plasma blood metabolites from migrating Garden Warblers recaptured during stopover on Ponza in relation to stopover duration (correlation statistics shown only when *P *<* *0.05). Minimal time on stopover (h) is the duration between recapture records. Intra-individual time series data were available from two individuals that were blood-sampled twice (filled diamonds with connected lines).

Antioxidant capacity and circulating uric acid of recaptured Garden Warblers did not change with minimum stopover time (*n* = 23, *r*_S_ = −0.018, *P *=* *0.935; and *n* = 23, *r*_S_ = −0.185, *P *=* *0.398, respectively) whereas oxidative damage significantly decreased with minimum stopover duration (*n* = 23; Fig.5). All three measures of fat metabolism were significantly correlated with minimum stopover duration, with *β*-hydroxybutyrate and nonesterified fatty acid levels decreasing (*n* = 22 and 16, respectively) and triglyceride levels increasing (*n* = 20) the longer birds were on the island before blood sampling (Fig.5).

## Discussion

Our analyses provide the first assessment of oxidative status in songbirds with different migration strategies early in their migration before an ecological barrier, as well as evidence that birds recover from oxidative damage on stopover after long flights. Our study provides evidence that birds build antioxidant capacity as they build fat stores at stopover sites before long flights, but does not support the idea that antioxidant stores remain elevated in birds with high fuel levels after an ecological barrier. We also show that birds on stopover are capable of recovering from the oxidative damage they have accrued during migration, as lipid oxidation levels decrease with time on stopover. Below we discuss these results as they relate to the four hypotheses tested, as well as the implications of our findings for understanding the effects of migration distance and body condition on the physiological strategies of songbirds.

### Hypothesis 1: birds prepare for migration by building antioxidant capacity and fat stores

Costantini et al. (2007:369) suggested that birds in “good condition,” that is, with high fuel stores, may have “reserves” of antioxidant capacity for controlling oxidative damage. Our study is the first to confirm this in free-living birds at a stopover site during migration. We found that antioxidant capacity and fat stores were positively correlated among both Blackpoll Warblers (fat score) and Red-eyed Vireos (fat mass) preparing for autumn migration at a northern stopover site, supporting the idea that birds build antioxidant capacity as they do energy stores before long-distance flights.

We expected Blackpoll Warblers to exhibit higher levels of antioxidant capacity than Red-eyed Vireos, given that the former would be preparing for an ocean crossing, but we did not find this to be the case. Rather, we found Blackpolls to have lower antioxidant capacity than vireos and significantly higher oxidative damage. This latter result would occur if the Blackpolls we captured had traveled a considerable distance to reach Block Island; Blackpolls that summer in Canada may travel up to 2500 km to reach staging areas in autumn on the east coast of the United States (Baird 1999), and evidence from several studies suggests that long flights increase oxidative damage (in pigeons, *Columba livia* Gmelin 1789, Costantini et al. 2008; in European Robins, *Erithacus rubecula* Linnaeus 1758, Jenni-Eiermann et al. 2014). The Red-eyed Vireos that we captured may have originated closer to Block Island than Blackpolls, and therefore sustained less oxidative damage before sampling.

### Hypothesis 2: birds use both antioxidant capacity and fat stores during migration

In contrast to Costantini et al.’s (2007) findings for Garden Warblers on Ponza in spring, we did not find circulating nonenzymatic antioxidant capacity to be correlated with fat score, fat mass, or muscle score in this study. Costantini et al. (2007) hypothesized that individuals with higher energy stores before long flights probably also have higher initial circulating nonenzymatic antioxidant levels, and therefore are better able – during the course of those flights – to prevent oxidative stress than leaner/antioxidant-poorer individuals, even when all flew the same distance. However, we have no evidence to support their suggestion that birds with higher energy stores after migration face lower oxidative stress by maintaining higher antioxidant reserves. Actually, the antioxidant capacity values in our study were considerably lower than what Costantini et al. (2007) documented and what we found in Blackpoll Warblers and Red-eyed Vireos; the oxidative damage values of Garden Warblers in our study were within the range of what we found for Block Island birds but higher than those reported by Costantini et al. (2007).

There are three possible explanations for why our results for Garden Warblers on Ponza differed from those of Costantini et al. (2007). First, the smaller sample size (<30 individuals per species) in the 2007 study may have not captured the variation seen in the current study with 129 Garden Warblers. Second, birds that we captured may have flown a longer distance before reaching the island, or encountered fewer antioxidant-rich foods than the Garden Warblers sampled by Costantini et al. (2007). Third, our sample was collected 10–20 days later in May when the pace of migration may be more intense with the approaching breeding season. For example, Wojciechowski et al. (2014) suggested that the nutritional priorities of Blackcaps (*Sylvia atricapilla* Linnaeus 1758) on stopover may change depending on season and the urgency of reaching the breeding grounds. Recent work with Hermit Thrushes (*Catharus guttatus* Pallas 1811) on Block Island (Smith and McWilliams 2014) has shown that the relationship between condition and stopover behavior changes as the fall migration season progresses. We suggest that Garden Warblers captured in mid-to-late May could present higher oxidative damage levels and less condition dependency in antioxidant stores than birds captured earlier in spring migration. Furthermore, the antioxidant status of birds, and the availability of dietary antioxidants, may vary considerably between fall and spring, given that the pace of migration is generally faster in anticipation of breeding, and the phenology of most temperate plants dictates that the peak abundance of antioxidant-rich foods is in autumn. This leads us to hypothesize that although birds may build antioxidant capacity as they do fuel stores on stopover, the accumulation or use of these antioxidants may depend not only on the duration of active flight but also on the pace of migration, including the length of stopover.

### Hypothesis 3: extent of circulating lipid oxidative damage is related to amount of lipids stored

We confirmed in all three species we examined that fat stores in birds at stopover sites were positively correlated with lipid oxidative damage. Stored fats are vulnerable to attack by pro-oxidants when protective mechanisms increase only linearly with fat mass; therefore, a high amount of stored fat can be expected to produce a high amount of lipid oxidation products (Pérez-Rodriguez et al. 2015). Jenni-Eiermann et al. (2014) found that oxidative damage to proteins (measured as protein carbonyls) did not correlate with fat, and so lipid stores or damage to those stores may not reflect the levels of oxidation products from other tissue precursors (e.g., flight muscle). We observed considerable variation in the range of damage experienced by the three species, with Red-eyed Vireos showing the least variability in damage levels; this pattern might be explained by a greater diversity of population origins of the longer distance Garden and Blackpoll Warblers at our study sites, although this explanation is only speculative and warrants further study with a greater suite of species and sites.

Many techniques are available for assessing oxidative status in animals (Costantini 2008; Monaghan et al. 2009), and controversy exists as to which measures, or combination of measures, may best represent an animal’s overall condition or oxidative state (Costantini and Verhulst 2009; Cohen et al. 2010; Hõrak and Cohen 2010). Lipids are particularly important molecules for migratory birds, and other animals that build large fat stores, for example, for migration or hibernation; and therefore, lipid oxidation products may continue to be a highly relevant measure of oxidative damage in bird studies. However, we urge future researchers to consider, when measuring circulating levels of lipid hydroperoxides, the importance of accounting for total mass or volume of fat in an animal as a whole.

### Hypothesis 4: birds recover both antioxidant and fat stores on stopover after a barrier

We collected two types of data to address how stopover duration after an ecological barrier affects migrating birds’ oxidative status and fat metabolism. Both yielded the same conclusions: Garden Warblers switched from fat catabolism to anabolism to recover their fat stores during stopover on Ponza, and lipid oxidative damage decreased, but circulating antioxidant capacity did not change with stopover length. Our limited repeated-measures data from two individuals sampled twice on stopover further confirmed these trends. Remarkably, oxidative damage levels plummeted in these two individuals within several days of stopping over, and interindividual data showed decreasing hydroperoxide levels over time, presumably as birds increased fat anabolism and replaced damaged lipids.

Interestingly, uric acid levels and antioxidant capacity were not associated with length of stay on Ponza, or with the differentiation between high-capture-day and low-capture-day birds. The absence of a trend in uric acid likely indicates that birds did not increase their intake of dietary protein on stopover, given that uric acid is the final product of protein catabolism in birds and correlates closely with protein consumption (Alan and McWilliams 2013). Alternatively, uric acid levels may have been similar between newly arrived birds and birds on stopover because the former catabolized body protein during flight and the latter catabolized dietary protein upon landing. The absence of a trend in antioxidant capacity with time on the island likely reflects the dearth of antioxidant-rich food resources in spring on Ponza. On Block Island and other fruit-rich stopover sites used in autumn, we suspect that birds increase their circulating antioxidant capacity as they consume antioxidant-rich fruits while stopping over, given that the autumn fruits birds consume most on Block Island tend to have the highest antioxidant content (Alan et al. 2013; Bolser et al. 2013). However, on Ponza and other fruit-poor stopover sites in spring, dietary antioxidants may be difficult to acquire.

## Conclusion

Our study is the first to show that songbirds build circulating antioxidant capacity as they do fat stores while preparing for migration and that flight distance before a stopover site may affect lipid oxidation levels. It does not, however, support the idea that antioxidant reserves remain in birds with high fuel stores after an ecological barrier. We further show that oxidative damage may be an inescapable hazard of using fats as the primary fuel for flight, but also that birds on stopover are capable of recovering from the oxidative damage they have accrued during migratory flight, as lipid peroxidation levels decrease with time on stopover. We further hypothesize that building circulating nonenzymatic antioxidant capacity on stopover likely depends on the resources available at the stopover site. Thus, the physiological strategy of migrating songbirds may be to build prophylactic antioxidant capacity in concert with fuel stores at stopover sites before a long-distance flight, and then repair oxidative damage while refueling at stopover sites after long-distance flight. The costs and benefits of long-jump versus short-hop migration strategies have been mainly derived from optimality models concerned with saving time, saving energy, or a combination of both (e.g., Hedenström and Alerstam 1997). We suggest that, in future, the ability of birds to prevent or repair oxidative damage resulting from sustained flight periods should also be considered in the context of migration ecology and evolution.

## Data Accessibility

Data are available from the Dryad Digital Repository: http://dx.doi.org/10.5061/dryad.sj6n2
